# 

**Published:** 1899-12

**Authors:** 


### Communicated.

### INTERNATIONAL DENTAL CONGRESS.

PARIS, FRANCE, AUGUST 8TH to 14TH, 1900.

The Committee appointed by the National Dental Association at the Omaha meeting, August 30th, 1898, was by order of the Chairman, Dr. A. W. Harlan, convened at the Cataract House, Niagara Falls, August 1st, 1899. No quorum being present, was adjourned to the 3rd inst. at 4 p. m.

There were then present: A. W. Harlan, Chicago; H. A. Smith, Cincinnati; Thomas Fillebrown, Boston; T. E. Weeks, Minneapolis; J. D. Patterson, Kansas City; H. W. Morgan, Nashville; T. W. Brophy, Chicago; W. C. Barrett, Baffalo; W. W. Walker, New York City; W. E. Griswold, Denver, Colo.; B. Holly Smith, Baltimore; J. Taft, Cincinnati, Ohio.

The meeting was called to order by the chairman, who gave a short address, stating the object, organization, etc., of the Congress, and the work necessary for the committee to accomplish in this country.

On motion of Dr. Weeks, W. E. Griswold was elected Secretary.

On motion of Dr. Fillebrown, Dr. Wm. Jarvie, of Brooklyn, N. Y., was elected an additional member of the committee.

On motion of Dr. Smith, the Chairman and Secretary were instructed to confer with the National Association in regard to arranging an earlier meeting next year to accommodate those going abroad.

On motion of Dr. Weeks, H. S. Sutphen, of Newark, N. J., was made a member of this committee.

On motion of Dr. Barrett, a place on this committee was reserved for the President of the National Association in the year 1900, and the chairman was authorized to insert his name.

On motion of Dr. Brophy, George H. Chance, of Portland, Oregon, was made a member of this committee.

On motion of Dr. Barrett, a resolution requesting any member of this committee, finding himself unable to go abroad to attend this Congress, to at once resign, and that the executive committee be empowered to fill the vacancy, was passed.

On motion of Dr. Smith, the chairman was requested to appoint a transportation committee composed of Dr. Jarvis, Dr. Walker, Dr. Harlan and Dr. Griswold.

On motion, a committee consisting of Dr. Brophy, Dr. Weeks, Dr. Morgan, was appointed to take charge of the exhibit of American Educational Methods.

On motion, an executive committee of five was appointed, consisting of Dr. A. W. Harlan, Chairman, Dr. Barrett, Dr. Brophy, Dr. E. C. Kirk and Dr. H. A. Smith.

On motion, the executive committee was empowered to fill vacancies in this committee.

On motion, adjourned to meet at call of chairman.

W. E. GRISWOLD, Secretary,
423 Mack Block, Denver, Colo.

EDITOR OF THE AMERICAN JOURNAL OF DENTAL SCIENCE.

Dear Sir;

The thirty fourth annual meeting of the Ohio State Dental Society will be held at the Grand Southern Hotel, Columbus, Ohio, December 5th, 6th and 7th, 1899. A good program consisting of essays and clinics has been prepared. A cordial invitation is extended to the profession at large.

HENRY BARNES,
Chairman Executive Committee.

---

EDITOR OF THE AMERICAN JOURNAL OF DENTAL SCIENCE.

Dear Sir:

The Pennsylvania Board of Dental Examiners will conduct examinations simultaneously in Philadelphia and Pittsburg, December 18th, 19th and 20th.

Application for examination must be made to the Hon. James W. Latta, Secretary of the Dental Council, Harrisburg, Pennsylvania.

G. W. KLUMP, Secretary,
Williamsport, Pa.

### To Readers and Correspondents.

Communications intended for publication, and exchange journals and papers, should be addressed to the Editor of The American Journal of Dental Science, No. 845 N. Eutaw St. Baltimore, Md. All letters relating to the business of the Journal, or containing cash remittances, to be directed to Snowden & Cowman Mfg. Co., 9 W. Fayette Street, Baltimore, Md. Articles for publication should be received by the Editor at least two weeks previous to the first of the month on which the number in which it is designed for them to appear is issued.

☞ The American Journal of Dental Science will contain fifty pages of reading matter in each number, making a volume of about Six Hundred pages,

EXCLUSIVE OF ADVERTISEMENTS.

